# Pre-harvest Sprouting and Grain Dormancy in *Sorghum bicolor*: What Have We Learned?

**DOI:** 10.3389/fpls.2018.00811

**Published:** 2018-06-15

**Authors:** Roberto L. Benech-Arnold, María V. Rodríguez

**Affiliations:** ^1^Cátedra de Cultivos Industriales, Departamento de Producción Vegetal, Facultad de Agronomía, Universidad de Buenos Aires, Buenos Aires, Argentina; ^2^Instituto de Fisiología y Ecología Vinculado a la Agricultura, Consejo Nacional de Investigaciones Científicas y Técnicas, Facultad de Agronomía, Universidad de Buenos Aires, Buenos Aires, Argentina; ^3^Cátedra de Fisiología Vegetal, Departamento de Biología Aplicada y Alimentos, Facultad de Agronomía, Universidad de Buenos Aires, Buenos Aires, Argentina

**Keywords:** *Sorghum bicolor*, grain sorghum, pre-harvest sprouting, seed dormancy, abscisic acid, dormancy QTL

## Abstract

The possibility of obtaining sorghum grains with quality to match the standards for a diversity of end-uses is frequently hampered by the susceptibility to pre-harvest sprouting (PHS) displayed by many elite genotypes. For these reasons, obtaining resistance to PHS is considered in sorghum breeding programs, particularly when the crop is expected to approach harvest maturity under rainy or damp conditions prevalence. As in other cereals, the primary cause for sprouting susceptibility is a low dormancy prior to crop harvest; in consequence, most research has focused in understanding the mechanisms through which the duration of dormancy is differentially controlled in genotypes with contrasting sprouting behavior. With this aim two tannin-less, red-grained inbred lines were used as a model system: IS9530 (sprouting resistant) and Redland B2 (sprouting susceptible). Redland B2 grains are able to germinate well before reaching physiological maturity (PM) while IS9530 ones can start to germinate at 40–45 days after pollination, well after PM. Results show that the anticipated dormancy loss displayed by Redland B2 grains is related reduced embryo sensitivity to abscisic acid (ABA) and increased levels of GA upon imbibition. In turn, transcriptional data showed that ABA signal transduction is impaired in Redland B2, which appears to have an impact on GA catabolism, thus affecting the overall GA/ABA balance that regulates germination. QTL analyses were conducted to test whether previous candidate genes were located in a dormancy QTL, but also to identify new genes involved in dormancy. These analyses yielded several dormancy QTL and one of them located in chromosome 9 (qGI-9) was consistently detected even across environments. Fine mapping is already in progress to narrow down the number of candidate genes in qGI-9.

## Introduction

Grain sorghum [*Sorghum bicolor* (L.) Moench] is a grass species cultivated for its grain which is used for feeding both humans and animals. Also known as *durra*, *jowari*, or *milo*, sorghum can grow under harsher conditions and therefore may be better than maize or sugarcane in some environments. Sorghum originated in Northern Africa but is now cultivated widely in tropical and subtropical environments to the point that it has become the fifth most important cereal of the world after rice, wheat, maize, and barley. Sorghum grains for human consumption are used to make flat breads and beverages and also for malting and brewery. As in many other crops, the sorghum grain is also required to perform as the propagule for a new crop. The possibility of obtaining grains with quality to match the standards for these end-uses is frequently hampered by the susceptibility to pre-harvest sprouting (PHS) displayed by many elite genotypes. Indeed, untimely germination in the mother plant promotes reserve mobilization and/or leads to either immediate loss of seed viability or to a large reduction in seed longevity (Del Fueyo et al., 2003). In addition, sorghum sprouts accumulate toxic amounts of cyanide (Ikediobi et al., 1988). For these reasons, obtaining resistance to PHS is one of the main objectives in sorghum breeding programs, particularly when the crop is expected to approach harvest maturity under rainy or damp conditions prevalence. As in other cereals, the primary cause for sprouting susceptibility is a low dormancy prior to crop harvest (see Rodríguez et al., 2015). Consequently, efforts have been directed to understand the mechanisms through which the duration of dormancy is differentially controlled in genotypes with contrasting sprouting behavior. In this review paper, we discuss the current understanding of the mechanisms that impose dormancy to the sorghum grain, and mention on-going work directed toward breeding for PHS tolerance.

## Where is Dormancy Located in the Sorghum Grain?

The sorghum grain is a caryopsis (Waniska and Rooney, 2002). Depending on the genotype, this caryopsis may be “naked” or covered to different extents by the hulls (consisting of the glumellae – lemma and palea – and the glumes). The grain comprises an embryo, reserve tissue (corneous and starchy endosperm, surrounded by the aleurone layer), and the seed coat (nucellus and testa) fused to the pericarp. As in other cereals, the external structures of the sorghum grain, including testa, pericarp, and hulls, can impose dormancy to the underlying embryo. These structures may accumulate various phenolic compounds, such as phenolic acids, coumarins, flavonoids, and tannins (Glennie, 1981; Dykes et al., 2009). Increased pigmentation has been frequently associated with deeper dormancy in cereals like rice, wheat, barley, and maize (as discussed in Rodríguez et al., 2015). Flavonoid compounds may have an inhibitory effect *per se*, as chemically active inhibitors of germination, or through an effect on permeability to gas exchange in the imbibed seed (as discussed in Rodríguez et al., 2015). Nevertheless, genetic studies have usually found that pigmentation-related loci that co-locate with loci for PHS or germination traits correspond with transcriptional regulators rather than structural genes involved in pigment biosynthesis. Red colored grains accumulate phlobaphenes or anthocyanins in the pericarp. Regulation of phlobaphene synthesis is exerted by R2R3-MYB transcriptional factors, as encoded by the *P1* gene in maize and *Yellow seed1* in sorghum (Kambal and Bate-Smith, 1976; Boddu et al., 2005; Ibraheem et al., 2015), whereas regulation of anthocyanin synthesis is controlled by transcriptional complexes consisting of R2R3-MYB, basic-helix-loop-helix (bHLH), and WD-repeat (WDR) proteins (MBW complex; Liu et al., 2015). Mutations in some of these genes can affect dormancy through pleiotropic effects on different pathways. In weedy red rice, SD7-1 (a BHLH-type regulator) activates simultaneously flavonoid and abscisic acid (ABA) synthesis genes in the developing seed, conferring red color and deep dormancy (Gu et al., 2011). Maize VIVIPAROUS-1 (VP1) is required for ABA signal transduction in the developing grain, and also regulates the expression of *C1* (encoding an R2R3 MYB factor) that promotes anthocyanin synthesis in the aleurone (McCarty et al., 1989). Loss-of-function alleles of *Tamyb10* (red pericarp, R-1 loci) in wheat and *Hvmyb10* in barley fail to accumulate proanthocyanidins in the testa and contribute to reduce grain sensitivity to ABA and dormancy (Himi et al., 2002; Himi and Noda, 2005). Sorghum grains can accumulate variable levels of flavonoids, including phlobaphenes in the outer pericarp and proanthocyanidines (condensed tannins) in the underlying testa (Cheng et al., 2009). Accumulation of tannins in the testa is independent of pericarp color and is controlled by Tannin1, a WD40-type transcriptional regulator (Wu et al., 2012). In a study with 42 sorghum hybrids by Harris and Burns (1970), a negative correlation between germination index and astringent tannin content was observed. Also, among tannin-less sorghums, red grained varieties are usually more resistant to PHS than genotypes with yellow and white grains, although no strict correlation has been demonstrated so far. A direct role for *Tannin1* and *Yellow seed1* in dormancy remains to be tested. Because condensed tannins are considered to reduce the nutritional value of the sorghum grain, research on PHS tolerance has focused on tannin-less sorghum varieties.

As reported for other cereals, the inability of the sorghum grains to germinate at harvest or before [*i.e.*, from 20 days after pollination (DAP) onward] is due to a complex interplay between coat- and embryo-based dormancy, with the former playing a major role in the overall contribution to grain dormancy. Removal of the covering structures (i.e., pericarp, testa and endosperm) results in a reduced expression of dormancy at a wide range of temperatures even with embryos isolated as early as 15 DAP (Benech-Arnold et al., 1991; Steinbach et al., 1995). However, different responsiveness to inhibitory factors such as ABA, as well as different accumulation of promoting factors such as gibberellins (GAs), support that embryo-related factors are relevant in the expression of different levels of coat-imposed dormancy.

## The System Redland B2 – IS9530

**FIGURE 1:**
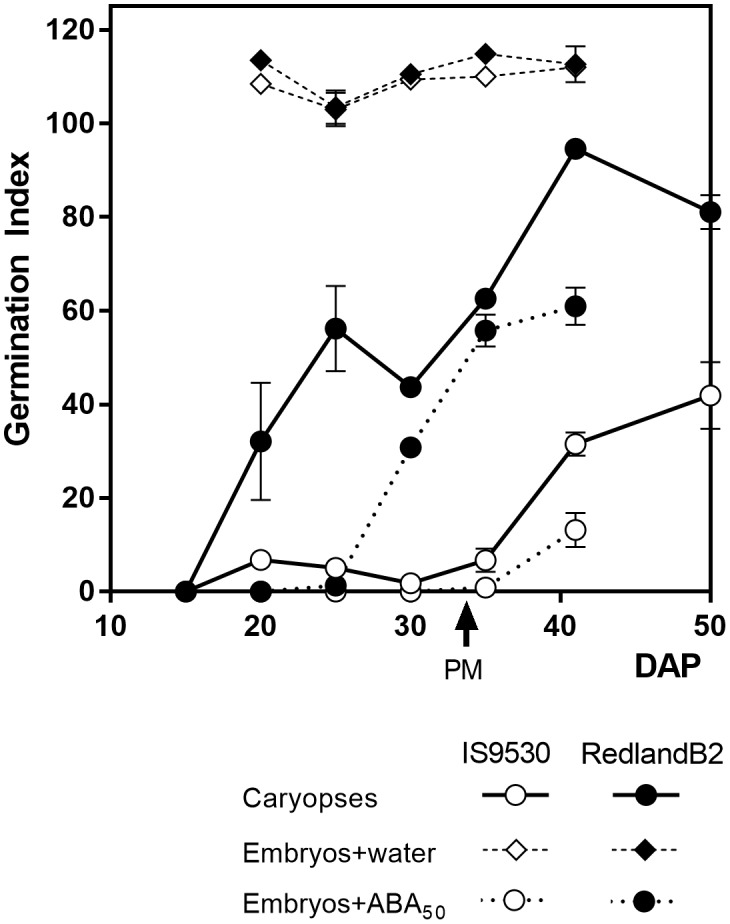
Germination index for sorghum lines IS9530 and RedlandB2. Caryopses were harvested on different days after pollination (DAP) and incubated for 12 days in water at 25°C (circles with solid lines). Embryos were dissected and incubated at 25°C in distilled water (diamonds) or 50 μM ABA (circles with dashed lines). Each data point is the mean value of two biological replicates (i.e., field plots) each one assessed in triplicate. Bars indicate SE of mean. Time of physiological maturity is indicated with an arrow. Experimental details are described in Rodríguez et al. (2009). Adapted from data first published by Rodríguez et al. (2009) and reproduced by permission of Oxford University Press (doi: 10.1093/aob/mcp184).

During the last 25 years, studies on the physiological and genetic bases of PHS resistance in sorghum were conducted using two inbred, red-grained lines as a model system: IS9530, a tall, tannin-less line derived from ICRISAT breeding program, and Redland B2 (originally registered as Redlan, or BTx378), an also tannin-less, three-dwarf derivative line, resulting from the US Sorghum Conversion Program (Stephens et al., 1967). During this program, sorghums were substituted with several height and maturity genes to obtain short, early forms in temperate zones. Despite their different background, both IS9530 and RedlandB2 display similar phenology at our latitude under sowing dates taking place between the last week of November and the first week of December. The sprouting behavior of these two inbred lines, however, is contrasting: the Redland B2 line is susceptible to PHS, while the IS9530 one is resistant. The nature of this contrasting behavior relies on the anticipated exit from dormancy of the Redland B2 grains which are able to germinate well before reaching physiological maturity (PM); IS9530 grains, in contrast, can start to germinate at 40–45 DAP, well after PM (**Figure 1**; Steinbach et al., 1995, 1997). Isolated embryos from both lines can germinate very quickly even before 20 DAP (**Figure 1**) demonstrating that the different capacity to germinate during development is the result of dormancy differentially imposed by the presence of the seed coat tissues. The pattern of dormancy release during grain filling normally shows two phases in Redland B2 grains: an early one which is not observed in IS9530 grains, starting around 15–20 DAP and reaching a plateau prior to PM (30–35 DAP), and a second one starting after PM and coinciding with the beginning of exit from dormancy in IS9530 grains (**Figure 1**). Susceptibility to PHS in Redland B2, then, is related to precocious grain dormancy loss, while resistance in IS9530 can be associated to a more persistent dormancy (Steinbach et al., 1995). It is well known, as in many other species, that the expression of dormancy in sorghum depends on the incubation temperature: dormancy is very much expressed at low incubation temperatures (i.e., 15–20°C), while it is barely expressed at high temperatures (i.e., 30–35°C; Benech-Arnold et al., 2003). For these reasons, differences in the sprouting behavior between these two lines are exacerbated when damp conditions prior to harvest are combined with mild ambient temperatures (20°C or below) that favor the expression of dormancy.

## Hormonal Regulation of Dormancy in the Developing Sorghum Grain

A crucial role for ABA in the imposition of physiological dormancy in the developing seed has been demonstrated in many species (Finkelstein et al., 2002). Mutant seeds of *Arabidopsis* and maize that are ABA-deficient or ABA-insensitive germinate precociously (Robichaud et al., 1980; Karssen et al., 1983). No sorghum mutants for ABA synthesis or metabolism have been reported, but application of the ABA-synthesis inhibitor fluridone during early seed development accelerated dormancy release in dormant IS9530 sorghum line (Steinbach et al., 1997), as expected from earlier experiments with fluridone that produced vivipary in maize (Fong et al., 1983). However, embryos from the more dormant line IS9530 were found to have a similar ABA content throughout development to embryos from the less dormant line Redland B2 (Steinbach et al., 1995). Embryonic content of ABA decreased gradually during phase two of imbibition, but this decrease was similar for both lines and did not relate with their germination response (Rodríguez et al., 2009). The seed coats were found to delay ABA leakage from the embryo to the medium, as ABA content decreased rapidly when isolated embryos were incubated in water (Gualano et al., 2007). This is due to the fact that ABA easily leaks from the naked embryo into the incubation medium, and suggests that the covers impose dormancy to the embryo, at least in part, by reducing ABA leakage. Instead, suppression of germination of Redland B2 embryos required ABA concentrations 10-fold higher than those required for inhibiting germination of IS 9530 embryos; moreover, this difference was maintained throughout the whole developmental period and even beyond PM (**Figure 1**; Steinbach et al., 1995). These results confirmed that the observed differences in responsiveness to ABA are due to functional differences in the ABA signaling pathway and not in embryonic ABA concentration (Gualano et al., 2007).

It has been known for a long time that GAs promote germination of dormant seeds in many species, antagonizing the inhibitory effect of ABA. Both ABA and GA also act antagonistically in the inception of dormancy during early development. Application of paclobutrazol, a GA biosynthesis inhibitor to young sorghum panicles, reduced GA content during development and delayed dormancy release for several weeks in Redland B2 grains (Steinbach et al., 1997). Moreover, the incubation of immature IS9530 grains coming from fluridone-treated panicles, in the presence of GA_4__+__7_, resulted in a pattern of exit from dormancy throughout development that resembled that of Redland B2 grains (Steinbach et al., 1997). Indeed, the first phase of dormancy release displayed by Redland B2 grains but not by IS 9530 ones (**Figure 1**), was mimicked by combining low ABA content with exogenous GA supplementation. These results suggested that the anticipated dormancy release displayed by Redland B2 grains was not only related to a low embryo responsiveness to ABA, but also to a high GA content or, alternatively, to a high capacity to synthesize GA *de novo* upon imbibition. Similar to observations of ABA content in developing grains of these two genotypes, no correlation was found between natural endogenous GA content and dormancy (Benech-Arnold et al., 2000). Instead, analysis of GA content in these same sorghum lines revealed that, in Redland B2, but not in IS 9530, GA_4_ content increased during imbibition and before completion of germination (Pérez-Flores et al., 2003). This increase in GA_4_ was directly related to a reduction in catabolite GA_34_ (Rodríguez et al., 2012) suggesting that GA catabolism has an active role in regulating GA_4_ levels. Taken together, these results suggest that the anticipated dormancy loss displayed by Redland B2 grains is related to a reduced embryo sensitivity to ABA and increased levels of GA upon imbibition. Therefore, both GA metabolism (in particular, GA catabolism) and ABA signaling appeared as the main components of the ABA/GA balance regulating the germination response in developing grains of both lines. A candidate gene approach was followed to assess and identify potential regulatory sites at the transcriptional level that would lead to the observed differences at the physiological level.

## Expression of Several ABA Signaling Genes and a GA Catabolism Gene is Upregulated in Imbibed, Dormant IS9530 Grains Before – but Not After – PM

Expression analyses were conducted for several candidate genes involved in ABA signaling and GA metabolism (Rodríguez et al., 2009, 2012). Orthologous sequences for the candidate genes in sorghum were obtained by searching the sorghum genome published by Paterson et al. (2009). Consistently with differences in sensitivity to ABA, a transient and coordinated up-regulation of *SbABI3/VP1*, *SbABI4*, *SbABI5*, and *SbPKABA1* (together with SbABI5 protein levels) occurred in imbibed grains of IS9530, but not in RedlandB2. This “induction” pattern in IS9530 was observed in immature grains (30 DAP), but after PM expression of these genes decreased rapidly and similarly in both lines (Rodríguez et al., 2009). This synchronous expression of main ABA signaling genes is in agreement with published results in Arabidopsis, where extensive cross-regulation has been demonstrated among VP1/ABI3, ABI4, and ABI5 (Lopez-Molina et al., 2002; Reeves et al., 2011).

Transcriptional analysis of several sorghum genes encoding putative GA synthesis enzymes (*SbEKO, SbEKAH, SbGA20ox2, SbGA20ox3*, and *SbGA3ox1*) showed, on the other hand, a transient increase in dormant grains (IS9530) during the first 2–3 days of grain imbibition, which did not occur in Redland B2 (Rodríguez et al., 2012). This evidence appears to be in contradiction with changes in GA_4_ levels in both lines. However, simultaneously with this enhanced expression of GA synthesis genes in dormant IS9530 grains, expression of GA inactivation genes *SbGA 2-oxidase1* and *Sb GA 2-oxidase3 (SbGA2ox1 and SbGA2ox3)* was also increased This observation, together with a negative association between embryo content of active GA_4_ and its corresponding catabolite GA_34_, supported the notion that GA_4_ levels remain low in dormant IS9530 grains as a result of a prominent catabolic activity by GA2-oxidases, which, in turn, is reduced in RedlandB2. On the other hand, incubation of dormant grains in 100 μM GA_3_ promoted germination but did not reduce the expression of most key GA synthesis genes, ruling out the idea of a negative feedback regulatory mechanism driven by active GAs. In addition, and in contrast to other reports which show a feed-forward mechanism affecting expression of *GA2-oxidase* genes by active GA levels in Arabidopsis (Ogawa et al., 2003; Rieu et al., 2008), expression of sorghum *SbGA2-ox1* and *SbGA2-ox3* was downregulated by exogenously applied GA_3_ (Rodríguez et al., 2012). The similar expression profiles obtained for *SbABI4*, *SbABI5* (together with SbABI5 protein abundance), and *SbGA2-oxidase* genes suggested the existence of a possible functional link between both pathways. This, together with the presence of an ABA-responsive complex (ABRC) conformed by *cis*-regulatory elements related to ABA (ABRE, RY repeat, and CE) in the 5′ regulatory region of *GA-2ox3*, suggested a possible interaction between ABA signaling pathway and GA catabolism (Rodríguez et al., 2009, 2012). This interaction was tested by Cantoro et al. (2013). In this work, SbABI4 and SbABI5 proteins were shown to interact *in vitro* with a fragment of the *SbGA2-ox3* promoter containing the above-mentioned ABRC. Both transcription factors were able to bind the promoter, although not simultaneously, suggesting that they might compete for the same *cis*-acting regulatory sequences. A biological role for these interactions in the expression of dormancy of sorghum grains was proposed: either SbABI4 and/or SbABI5 activate transcription of the *SbGA2-oxidase3* gene *in vivo* and promote SbGA2-oxidase3 protein accumulation; this would result in active degradation of GA_4_, thus preventing germination of dormant grains. A comparative analysis of the *5′*-regulatory region of *GA2-oxidase* genes from both monocots and dicots showed that *GA2-oxidase* genes with an ABRC in their promoter region are exclusive to a conserved sub-group found in monocots (sub-group M3, according to Cantoro et al., 2016) to which *SbGA2-ox3* belongs. Conservation of the ABRC in closely related GA2-oxidases from *Brachypodium distachyon* and rice suggests that these species might share the same regulatory mechanism as proposed for grain sorghum.

In summary, physiological evidence (showing similar endogenous ABA levels but contrasting response to exogenous ABA) and transcriptional data (showing different expression levels of ABA signaling genes but also of ABI5 protein levels) support that ABA signal transduction is impaired in Redland B2. Reduced ABA signaling appears to have an impact on GA catabolism, thus affecting the overall GA/ABA balance that regulates germination.

## Genetic Approach: Mapping of “Old” Candidate and “New” Dormancy Genes

**FIGURE 2:**
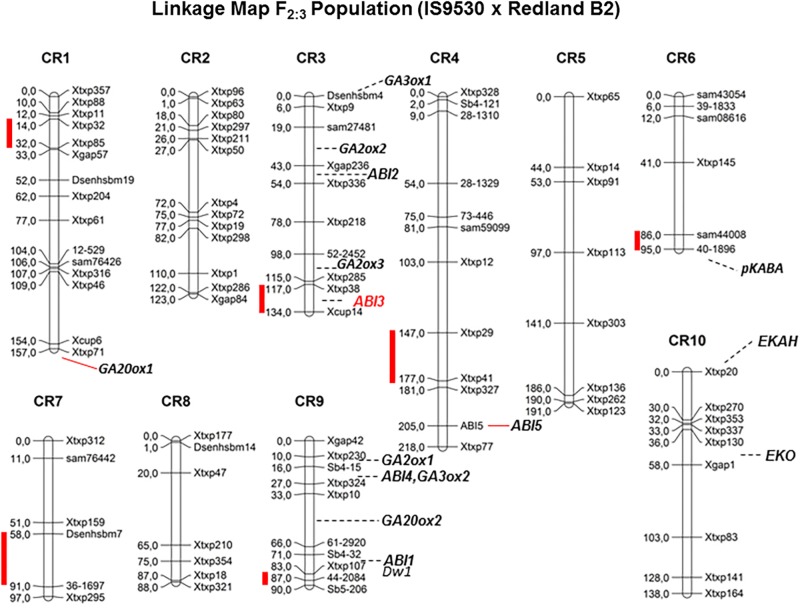
Genetic linkage map for F2 grain sorghum population, derived from IS9530 and RedlandB2 inbred lines, built with 96 SSRs segregation analysis (Adapted from Cantoro et al. (2016), by permission from Springer, Euphytica, ^®^2016). Red bars indicate position of dormancy related QTL as reported by Cantoro et al. (2016). Marker names are shown on the right side of each chromosome and genetic distances between markers (in cM) are indicated on the left side. Positions of *Dwarf1*, and ABA signaling and GA metabolism candidate genes are shown.

Quantitative trait loci (QTL) analyses were conducted to investigate the genetic bases of the contrasting dormancy phenotypes in both sorghum inbred lines IS 9530 and Redland B2. This approach would allow testing whether previous candidate genes are located in a dormancy QTL, but also would lead to the identification of new genes involved in dormancy. The first genetic study was done by Lijavetzky et al. (2000) with an F_2_ mapping population derived from a cross between IS9530 x Redland B2. These authors used RFLP, RAPDs, and AFLP markers, and reported a putative association between *SbVP1* and a dormancy QTL in likage group “E” (Carrari et al., 2003). To further confirm and expand these results, a new mapping population was obtained from the same sorghum parents, the genetic map was constructed using SSR markers, and phenotypic data were obtained at 34 (before PM) and 45 (after PM) DAP (Cantoro et al., 2016). In this work, six QTLs were identified for seed dormancy (**Figure 2**) in 42 DAP grains (qGI-1, qGI-3, qGI-4, qGI-6, qGI-7, and qGI-9) which were successfully anchored on the *S. bicolor* genome assembly v2.1 (Paterson et al., 2009) through QTL flanking SSR physical position. The number of genes within these intervals ranged from 75 (*qGI-9*) to 547 (*qGI-3*). No epistasis was detected for the identified QTL. Interestingly, *SbABI3/VP1* located within qGI-3, which makes it a good candidate to be the causal gene for this QTL. Allelic variants for *SbVP1* were found to exist in RedlandB2 and IS9530 (Carrari et al., 2001). The sorghum *VP1* gene encodes a 699 amino acid predicted protein, and sequences obtained for RedlandB2 and IS9530 differ in two residues at positions 341 (Gly/Cys, within the repression domain) and 448 (Pro/Ser; Carrari et al., 2001). These replacements might result in different biological functions, particularly the one located in the repression domain (Hoecker et al., 1995). Interestingly, in Arabidopsis seeds of *abi3* mutants, ABI5 expression was greatly reduced (Lopez-Molina et al., 2002), while ectopic expression of maize *VP1* in Arabidopsis *abi3* background inhibited *ABI1/ABI2* induction by ABA (Suzuki et al., 2003). An altered functionality of Redland B2 *SbVP1* allele appears as a possible cause for the altered expression of other ABA signaling genes as observed in imbibed, developing grains. Future experiments will test whether sorghum allelic variants for *SbVP1* have different activation/repression activity, together with fine mapping for this QTL. In parallel, synteny analysis of all detected QTL was also carried out and, besides *VP1*, no previous dormancy-related *loci* reported in other cereals were found to locate with sorghum QTLs reported by our group (Cantoro et al., 2016).

Ongoing work relies on a recombinant inbred line (RIL) population derived from the F_3_ mapping population used by Cantoro et al. (2016). This population was phenotyped recently for several dormancy-related traits, including sprouting in the field which occurred naturally in 2016. A new set of SNP markers was used, and due to an unbalanced coverage in several chromosome regions, QTL detection was limited to chromosome 9. Using two-year phenotypic data obtained before and after PM, qGI-9 (as reported by Cantoro et al., 2016) was identified again in this new RIL population. Improved marker coverage is expected to allow detection of previous dormancy QTLs including qGI-3. Interestingly, the region containing the dormancy *locus* in qGI-9 is very close to *DWARF-1*, a gene regulating plant stature and recently cloned by two groups (Hilley et al., 2016; Yamaguchi et al., 2016). Sorghum DW1 protein positively regulates brassinosteroid (BR) signaling and plant stature by inhibiting a negative regulator of BR signaling, BIN2 (Hirano et al., 2017). The genomic region in chromosome 9 carrying the loss-of-function dw1 allele has been introduced into many elite lines during the Sorghum Conversion Program to reduce plant stature, including RedlandB2 and BTx623. As the Standard Yellow Milo background (where the *dw1* mutation originated; Quinby and Karper, 1961) displays very low dormancy prior to harvest, it is likely that a low-dormancy allele was introduced together with the *dw1* allele during breeding for reduced height. Low heterozygocity along this region of chromosome 9 in a large GWAS panel (Morris et al., 2013) suggests that, if both *dw1* and the qGI-9 causal gene are linked, then many elite parental lines harbor this low dormancy allele. Current work involves the obtention of NILs for fine mapping of both qGI-3 and qGI-9 and genotyping by sequencing (GBS) of parental lines. Both strategies are aimed to narrow down the number of candidate genes in these regions and identify DNA polymorphisms in these genes. Future studies including transcriptomic analysis of developing grains for both parental lines are expected to help identify potential transcriptional variants (due to polymorphisms in the promoter region) among the candidate genes within each QTL. Strategies based on the combination of genetic and genomic tools have accelerated the process leading to identify a solid, causal gene for a QTL. An example of this is the identification of MOTHER OF FT (MFT) as the causal gene of a dormancy QTL (QPhs.ocs-3A.1) in wheat by Nakamura et al. (2011). Confirmation of the role of the proposed candidate genes for qGI-9 and qGI-3 in the dormancy phenotype will require functional testing either in sorghum or in other model organisms.

## Author Contributions

All authors listed above made a substantial, direct and intellectual contribution to this work. RB-A and MR discussed the contents and wrote the first version of the manuscript. MR corrected the revised version. Both authors read and approved the final version for publication.

## Conflict of Interest Statement

The authors declare that the research was conducted in the absence of any commercial or financial relationships that could be construed as a potential conflict of interest.
